# Bridging length scales in hard materials with ultra-small angle X-ray scattering – a critical review

**DOI:** 10.1107/S2052252524006298

**Published:** 2024-08-01

**Authors:** Fan Zhang, Jan Ilavsky

**Affiliations:** a Materials Measurement Science Division National Institute of Standards and Technology 100 Bureau Drive Gaithersburg Maryland20899 USA; b X-ray Science Division, Advanced Photon Source Argonne National Laboratory Lemont IL60439 USA; ESRF, France

**Keywords:** ultra-small angle X-ray scattering, USAXS, hard materials, microstructures, *in situ* characterization

## Abstract

Owing to their exceptional properties, hard materials such as advanced ceramics, metals and composites have enormous economic and societal value, with applications across numerous industries. Understanding their microstructural characteristics is crucial for enhancing their performance, materials development and unleashing their potential for future innovative applications. However, their microstructures are unambiguously hierarchical and typically span several length scales, from sub-ångstrom to micrometres, posing demanding challenges for their characterization, especially for *in situ* characterization which is critical to understanding the kinetic processes controlling microstructure formation. This review provides a comprehensive description of the rapidly developing technique of ultra-small angle X-ray scattering (USAXS), a nondestructive method for probing the nano-to-micrometre scale features of hard materials. USAXS and its complementary techniques, when developed for and applied to hard materials, offer valuable insights into their porosity, grain size, phase composition and inhomogeneities. We discuss the fundamental principles, instrumentation, advantages, challenges and global status of USAXS for hard materials. Using selected examples, we demonstrate the potential of this technique for unveiling the microstructural characteristics of hard materials and its relevance to advanced materials development and manufacturing process optimization. We also provide our perspective on the opportunities and challenges for the continued development of USAXS, including multimodal characterization, coherent scattering, time-resolved studies, machine learning and autonomous experiments. Our goal is to stimulate further implementation and exploration of USAXS techniques and inspire their broader adoption across various domains of hard materials science, thereby driving the field toward discoveries and further developments.

## Abstract 2

This review examines the use of ultra-small angle X-ray scattering (USAXS), a nondestructive technique for analyzing the multi-scale microstructures of hard materials such as ceramics, metals and composites. It discusses the principles, benefits and challenges of USAXS, along with its potential to advance materials development and optimize manufacturing processes, while also considering future enhancements through multimodal characterization and machine learning.

## 1. Introduction

The development of materials science over the last century has profoundly transformed the world. Its impact reaches every corner of modern life, from the electronic components in smartphones to the essential materials used by the aerospace industry in humanity’s pursuit of becoming a multi-planetary species. Understanding, controlling and harnessing materials structure are at the heart of this development because the structure – be it atomic, mesoscopic, microscopic or macroscopic – dictates almost every aspect of materials properties and performance. Significant developments in the structure characterization techniques and instruments such as electron microscopy, X-ray scattering, diffraction and spectroscopy; atomic force spectroscopy; nuclear magnetic resonance spectroscopy; and neutron scattering and diffraction have played a foundational role in driving technological innovations in materials science.

Materials structures are complex. A wide range of materials have hierarchical structures (Yang *et al.*, 2017 ▸, Juarez *et al.*, 2017 ▸; Li *et al.*, 2016 ▸). In these materials, atoms are organized at several distinct length scales, typically ranging from nanoscales to microscopic or even macroscopic scales. The structural hierarchy at different length scales is interdependent, and such interplay contributes to the overall properties and behaviors of the materials (Carpinteri & Pugno, 2008 ▸), making its proper understanding crucial. Notably, hierarchical materials are found extensively in nature (Fratzl & Weinkamer, 2007 ▸). Materials such as bones, wood, spider silk, sponges, tooth enamel and feathers, all with their exceptional properties such as strength and flexibility, clearly demonstrate the evolutionary advantage of hierarchical materials. The structure hierarchy is also ubiquitous in modern engineered materials (Parlett *et al.*, 2013 ▸; Kim *et al.*, 2015 ▸). For example, structural materials have an enormous impact on the world economy. Without exception, the primary structural materials we use today, such as steel, cement, ceramics and composites, all possess hierarchical structures. With the advancement of computational materials science, exploiting this complex structural organization over different length scales has increasingly become a principal component of materials design and optimization to meet major societal needs such as sustainability (McDowell & Olson, 2009 ▸).

**Figure 1:**
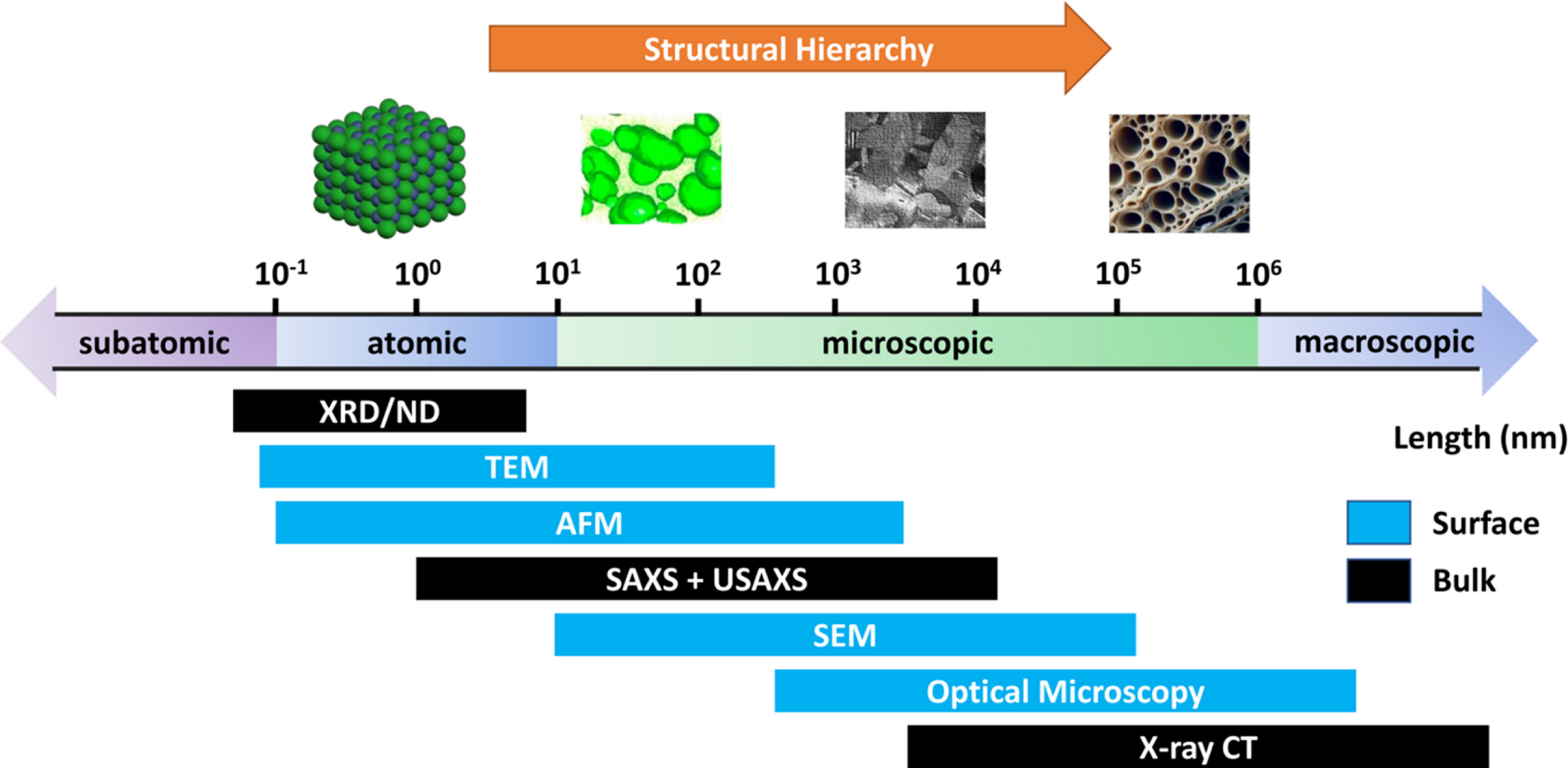
Typical length scales in the materials science of hard materials. The structures above the length scale arrow (from left to right) are the crystal structure, precipitates, grain structure and meso-structure. The analytical techniques for structural measurements shown below the arrow are color-coded for surface and bulk methods. From top to bottom, they are X-ray diffraction and neutron diffraction (XRD/ND), transmission electron microscopy (TEM), atomic force microscopy (AFM), small-angle X-ray scattering and ultra-small-angle X-ray scattering (SAXS/USAXS), scanning electron microscopy (SEM), optical microscopy, and X-ray computed tomography (X-ray CT).

Characterization of hierarchical materials can be a challenging task due to the length scales involved (Dingreville *et al.*, 2016 ▸). Many of the advanced structure characterization techniques have their optimal length scale ranges – an example is shown in Fig. 1 ▸. For instance, transmission electron microscopy provides exceptional insight into the chemical and spatial details of atomic arrangements. However, they are limited in both the lateral and the transverse dimensions of the observation window, induced by the focal conditions of the electron beams and the electron-matter interactions (Williams *et al.*, 1996 ▸). Conversely, X-ray computed tomography, a widely used nondestructive technique to probe 3D structures, has a resolution limit at several micrometres for in-house equipment, dictated by parameters such as detector pixel size and X-ray beam focal conditions (Withers *et al.*, 2021 ▸). In contrast, dedicated synchrotron based microtomography instruments can achieve a resolution of approximately 1 µm with fields of view (FOV) exceeding several millimetres (Fusseis *et al.*, 2014 ▸). State-of-the art synchrotron based nanotomography setups can achieve resolutions from sub-10 nm to a few tens of nanometres but with a limited FOV of micrometres (Michelson *et al.*, 2022 ▸; Cao *et al.*, 2020 ▸). This trade-off between resolution and FOV arises from factors such as optical and detector limitations, beam intensity and coherence, and positioning stability. Additionally, with its energy tunability, synchrotron based tomography can provide elemental sensitivity, enabling 3D chemical mapping. Overwhelmingly, the characterization of materials with hierarchical structures relies on the combined applications of several techniques, each providing insight on a specific scale (Mitchell *et al.*, 2015 ▸). This piecemeal approach means that correlating data across different scales is challenging, leading to difficulties in constructing a cohesive understanding of the materials’ behaviors.

Such challenges are exacerbated when understanding a material’s response to external stimuli is needed. Frequently, these responses are kinetics-driven, instead of thermodynamics-driven, meaning that a materials’ behaviors are governed by the rate of the underlying processes instead of the equilibrium state they reach. The kinetic aspects of these processes demand ‘nondestructive’ measurements of the time-dependent changes in the materials’ structures. Even if the processes are completely reproducible, which is normally a strong assumption, measurements with different techniques would nevertheless be subject to their different time resolutions and differences in sample volumes. Microscopic and spectroscopic techniques tend to be surface-sensitive while diffraction techniques are bulk-sensitive. This added layer of complexity renders it even more difficult to apply a jigsaw puzzle approach.

To overcome these challenges, over the last four decades, the technique of USAXS was developed, primarily at synchrotron sources (Narayanan *et al.*, 2018 ▸; Ilavsky *et al.*, 2009 ▸; Konishi *et al.*, 1997 ▸) and neutron facilities (Agamalian *et al.*, 1998 ▸; Jericha *et al.*; 2007 ▸; Rehm *et al.*, 2013 ▸), and more recently by commercial vendors in the form of in-house equipment with the improvement of X-ray sources, optics and detectors. The original design focused on the application of Bonse–Hart interferometer-type crystal optics (Bonse & Hart, 1965*a* ▸), which provides the required resolution in reciprocal space to probe microstructures in the size range from tens of nanometres to several micrometres. More recently, the increase in beam flux and improvements in hardware, together with the incorporation of complementary techniques, have allowed Bonse–Hart USAXS instruments to access a continuous size range from sub-ångstrom to several tens of micrometres (Van Vaerenbergh *et al.*, 2016 ▸; Ilavsky *et al.*, 2018 ▸). Concurrently, there is a worldwide pursuit of long small-angle scattering beamlines in synchrotron facilities (Narayanan *et al.*, 2022 ▸), leading to a maximum measurable size exceeding micrometres under specific operating conditions. In the more recent past (last two decades), the temporal resolution required to understand materials kinetics has also drastically improved for facility based USAXS instruments. Together, these improvements in spatial size range and temporal resolution make USAXS a powerful nondestructive technique for evaluating material structures, especially for those with hierarchical structures.

Even though in-house instruments have become available, the field of USAXS is still predominantly user-facility centered. This article focuses on the state of the field of USAXS and critically evaluates the advantages and disadvantages of different types of USAXS instruments. Our review is focused on the application and opportunities for hard materials, *i.e.* materials characterized by their rigidity and resistance to deformation, such as metals, ceramics and geological materials. In addition to their economic value, these materials present additional challenges for structural characterization owing to their high electron density. Unlike soft materials such as polymers and colloids, where the USAXS applications have been described in several review articles (Bhatia, 2005 ▸; Zhang & Ilavsky, 2010 ▸), a critical review of USAXS focused on hard materials is lacking, though the research opportunities are plenty. We hope that this review will provide a comprehensive overview and inspire further research and development in the application of USAXS in the study of hard materials. Our emphases are to highlight the unique aspects of USAXS when applied to hard materials, discuss the specific challenges these materials present, showcase how USAXS has been successfully used to overcome these challenges and provide our perspectives on future opportunities. Through this review, we aim to bridge the gap in the literature and provide a valuable resource for researchers and practitioners working with hard materials.

## 2. USAXS instrumentation

For readers unfamiliar with USAXS, a natural first question is: what does ‘ultra’ mean in USAXS? ‘Ultra’ is a descriptive term and, as such, does not have a formal definition recognized by the International Union of Crystallography, the global governing body for crystallography. However, over the past decades, the small-angle scattering community has generally agreed that USAXS measures scattering inhomogeneities larger than a micrometre. In this review, we will use the formula *D* = 2π/*q*_min_ to estimate the size of the scatterer, where *D* represents the size of the scattering object and *q*_min_ is the minimum value of the scattering vector *q* magnitude. Here, *q* = 4π/λsin(θ), where λ is the X-ray wavelength and θ is one half of the scattering angle 2θ.

When considering USAXS studies of hard materials, two primary factors come into play. The first factor is related to the instrumentation itself, whereas the second factor concerns the characteristics unique to hard materials. These two factors will be discussed separately.

### 2.1. Two types of USAXS instrumentation

A USAXS measurement needs to probe the USAXS size regime effectively. To measure sizes up to the micrometre scale, *q*_min_ must be lower than 0.001 Å^−1^. Furthermore, the resolution of the measurement (Δ*q*), which refers to the range of the *q* values integrated together for a data point (scattering intensity at a given *q* value), should be on the order of *q*_min_. This condition is necessary to ensure that a sufficient number of meaningful data points are collected below 0.001 Å^−1^ so that the scattering profile can be modeled with confidence. For ease of discussion, we will adopt a general guideline: *q*_min_ should be 0.0006 Å^−1^ or smaller for an instrument to be considered USAXS. Additionally, the chosen Δ*q* should enable the instrument to collect at least five data points, though preferably ten or more, for *q* values below 0.001 Å^−1^. This guideline ensures a detailed and accurate characterization of the scattering in the USAXS region.

**Figure 2:**
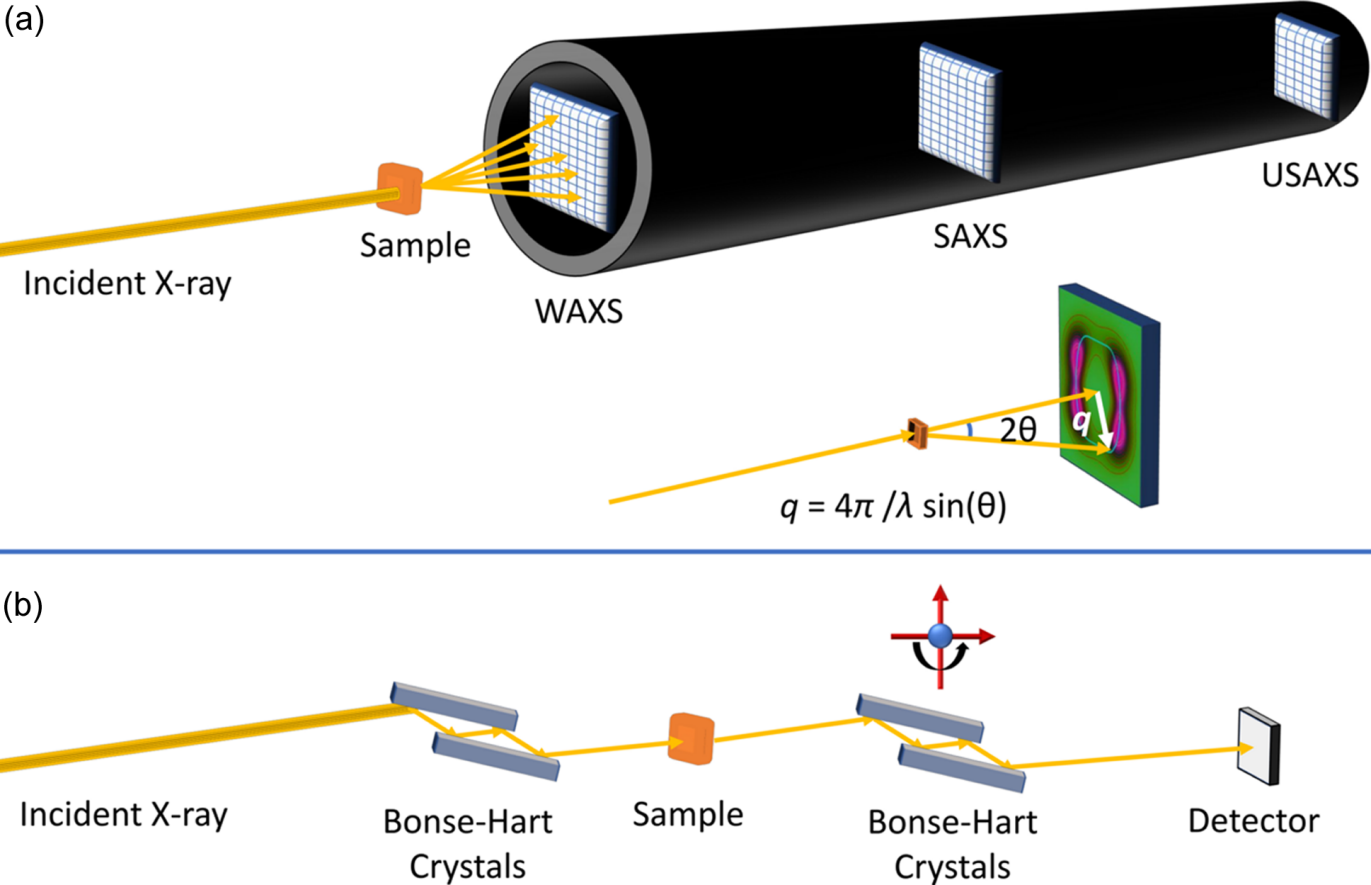
Schematics of two primary types of USAXS Instruments. (*a*) Pinhole configuration: in this setup, the scattering pattern is typically recorded on a 2D area detector. USAXS data are generally collected using the maximum feasible sample-to-detector distance, contingent on the specific sample-to-detector distance and the X-ray wavelength. (*b*) Bonse–Hart Type USAXS instrument: the *q* resolution depends on the crystal optics, the order of reflection and the X-ray wavelength.

To meet these requirements, two primary designs for USAXS instruments exist, with their schematics shown in Fig. 2 ▸. The first type, illustrated in Fig. 2 ▸(*a*), is a pinhole SAXS instrument. Here, after scattering from a sample, X-rays are captured by a 2D area detector. The pinhole SAXS instrument is the most widely used worldwide, with most synchrotron facilities today offering one or more SAXS devices in this configuration. Some of these instruments are capable of accessing the USAXS regime, although often under highly specific operational conditions such as with low X-ray energies. These instruments are typically equipped with area detectors with pixel sizes smaller than 100 µm (*e.g.* Eiger^1^ from Dectris), which is beneficial for USAXS because small pixels, with their small solid angles, improve the *q* resolution required for USAXS. X-rays are focused on the detector plane, as opposed to focusing on the sample plane, to reduce the footprint of the incident X-ray beam on the detector and allow the detector to access the smallest possible *q*. These instruments typically have a long flight tube that allows for a sample-to-detector distance exceeding 8 m. The beamstop and other optical elements that can introduce parasitic scattering also need to be carefully configured for the data to qualify as USAXS. Even with a flight tube length between 8 and 10 m, an X-ray energy below 8 keV will be required to meet the USAXS definition. This low X-ray energy requirement, as detailed in the subsection below, makes such instruments difficult to utilize with hard materials. For pinhole instruments to access the USAXS range at sufficiently high energy (20 keV and higher), a longer flight tube, potentially 20 m or more, would be required.

Facility based SAXS instruments are often designed and configured to meet the primary needs of their respective user communities. Although solid-state phase transformations in alloys were among the first applications of SAXS (Guinier, 1938 ▸), the flourishing of SAXS as a technique in today’s materials science would not be possible without generations of soft-material scientists who see the value of SAXS in characterizing nanoscopic and mesoscopic structures of a broad range of materials, such as polymers and colloids (Pedersen, 1997 ▸, Ballauff, 2001 ▸) that have feature sizes on the orders of nanometres and above. These materials often do not possess long-range order, making methods such as X-ray diffraction less effective. Because of this, many of the existing SAXS instruments are best suited for characterizing soft materials or materials where sample transmission is typically not a concern. When used at higher energies, their *q* range would be reduced, making larger scattering features in hard materials inaccessible.

The characteristics of the pinhole camera are widely known. For brevity, we will not enumerate these characteristics here. Instead, we will compare the critical aspects of two types of USAXS designs in a later section.

The second design is based on Bonse–Hart type optics. A schematic of a Bonse–Hart USAXS device is shown in Fig. 2 ▸(*b*). Bonse–Hart devices, designed for either X-rays or neutrons, use a specialized setup of analyzer crystals, known as ‘channel-cut’ crystals, to measure the intensity of the beam scattered from a sample. These ‘channel-cut’ crystals allow for multiple Bragg diffractions from single crystals, thereby selecting an extremely angularly narrow beam based on its angular (or reciprocal) space position.

Such a setup typically involves two pairs of crystals: the first pair, called collimating crystals, is positioned before the sample to precisely collimate the incoming beam. The second pair, known as analyzer crystals, is located after the sample to ‘analyze’ or measure the scattered intensity. Both crystal sets utilize Bragg diffraction where, according to dynamic diffraction theory, the width of the crystal diffraction curve (rocking curve) becomes exceptionally narrow (Δ*q*/*q* is ≃ 10^−4^ or smaller). Employing multiple diffractions within the channel-cut crystals further enhances this effect, reducing the intensity of the rocking curve tail exponentially (Bonse & Hart, 1965*b* ▸) and thus enhancing the Bonse–Hart instruments’ bandwidth selectivity (angular resolution). During measurements, the collimating crystals are fixed, while the analyzer crystals rotate with precision to selectively diffract X-rays scattered at minimal angles from the incident beam. This method effectively filters out any unscattered or broadly scattered X-rays, ensuring highly accurate and detailed measurement of the scattering profile.

Unlike the pinhole camera, Bonse–Hart USAXS instruments are less common and require further description. They possess the following specific features:

(1) The *q* resolution is a function of the crystal optics. The typical optics available today include Si(111) with *q*_min_ ≃ 1.2 × 10^−4^ Å^−1^ and Si(220) with *q*_min_ ≃ 0.8 × 10^−4^ Å^−1^. In the future, with improvement of instrumental stability, it is expected that Si(440) and Si(660) optics, with their *q*_min_ values at 0.3 × 10^−4^ Å^−1^ and 0.1 × 10^−4^ Å^−1^, respectively, will provide the possibilities to further expand the Bonse–Hart instrument accessible *q* range to tens of micrometres.

(2) The intensity range and data quality depend heavily on the available X-ray flux, because of the small angular acceptance window of the crystal optics and the brief measurement duration related to point scanning. This is especially true for scattering intensity measurement at higher *q* values (*q* > 0.01 Å^−1^), as the intensity of small-angle scattering usually diminishes rapidly according to a power-law decay with an increase in *q*.

(3) The *q* resolution of the instrument is independent of beam size, making large beam measurements of statistically averaged scattering features routine.

(4) Focusing X-rays on the sample or detector is generally counterproductive, since the collimating channel-cut only allows the parallel part of the incoming beam to pass through.

(5) A mismatch between the diffraction planes of the Bonse–Hart crystals and the beamline monochromators removes the need for harmonic rejection mirrors (Zhang, Allen *et al.*, 2018 ▸). This removal of harmonic rejection mirrors also helps to better preserve the spatial coherence of the X-ray beam.

Note that Bonse–Hart devices are compact and can easily be accommodated within a space occupying less than 1.5 × 2.5 × 1.5 m. This compactness is significant, as pinhole instruments equipped with long flight tubes and large area detectors are considerably more expensive, often costing an order of magnitude or more than a Bonse–Hart device.

There are two approaches to designing USAXS/SAXS instruments incorporating Bonse–Hart optics. The first approach involves adding the Bonse–Hart device to an existing pinhole instrument. This allows for the measurement of a smaller *q* range specific to the ultra-small angle regime, while utilizing a wide-range SAXS instrument for scattering at higher angles (*q* values). The advantage of this approach lies in the relative simplicity of the Bonse–Hart components’ design and the smaller, simpler crystal optics required. For measurements, this device is positioned in front of and behind the sample, typically within a vacuum chamber. This design is featured in recent commercially available desktop devices, such as those by Anton–Paar and Xenocs, and is also being implemented at Diamond Light Source, UK, for the I22 beamline (Pauw *et al.*, 2021 ▸). The USAXS capability of the Diamond setup covers the *q* range 0.00015–0.02 Å^−1^, allowing for easy and rapid interchange between SAXS and USAXS measurements by shifting the modular components on their motion stages. The use of vertical crystal rotation axes simplifies construction with minimal impact on efficiency. Routine implementation of this device is currently in progress.

The second option is a larger Bonse–Hart device capable of measuring both USAXS and a significant portion of the SAXS *q* range using crystal optics. This design, exemplified by the APS Bonse–Hart USAXS instrument available since 1999 (Ilavsky *et al.*, 2018 ▸, 2009 ▸), benefits from Bonse–Hart theoretical ability of the geometry to measure a wide range of angles. This is akin to analyzer based high-resolution powder diffraction instruments found at synchrotrons (Lee *et al.*, 2008 ▸; Fitch, 2004 ▸). Such an instrument also has the flexibility to perform 2D collimated USAXS measurements when needed (Ilavsky *et al.*, 2002 ▸).

The APS USAXS instrument has been successfully tested and utilized for measurements up to *q*_max_ ≃ 1 Å^−1^, though at present is operated up to *q*_max_ = 0.3 Å^−1^. To measure scattering intensity at higher *q* values, an additional small pinhole SAXS camera is used. Owing to its broad *q* range, this Bonse–Hart USAXS instrument can operate independently for tasks not requiring *q* values higher than 0.3 Å^−1^, thus speeding up data collection. Nonetheless, incorporating additional SAXS and wide-angle X-ray scattering (WAXS, *i.e.* XRD) devices proves beneficial for extending the *q* range. These devices not only offer greater sensitivity but also enhance the overall intensity range of the collected data. In such configurations, data are gathered sequentially through any combination of these three techniques. When all devices are employed in concert, the integrated USAXS/SAXS/WAXS system provides a comprehensive *q* range spanning nearly five decades, from 0.0001 to over 6 Å^−1^.

### 2.2. X-ray energy consideration for hard materials

**Figure 3:**
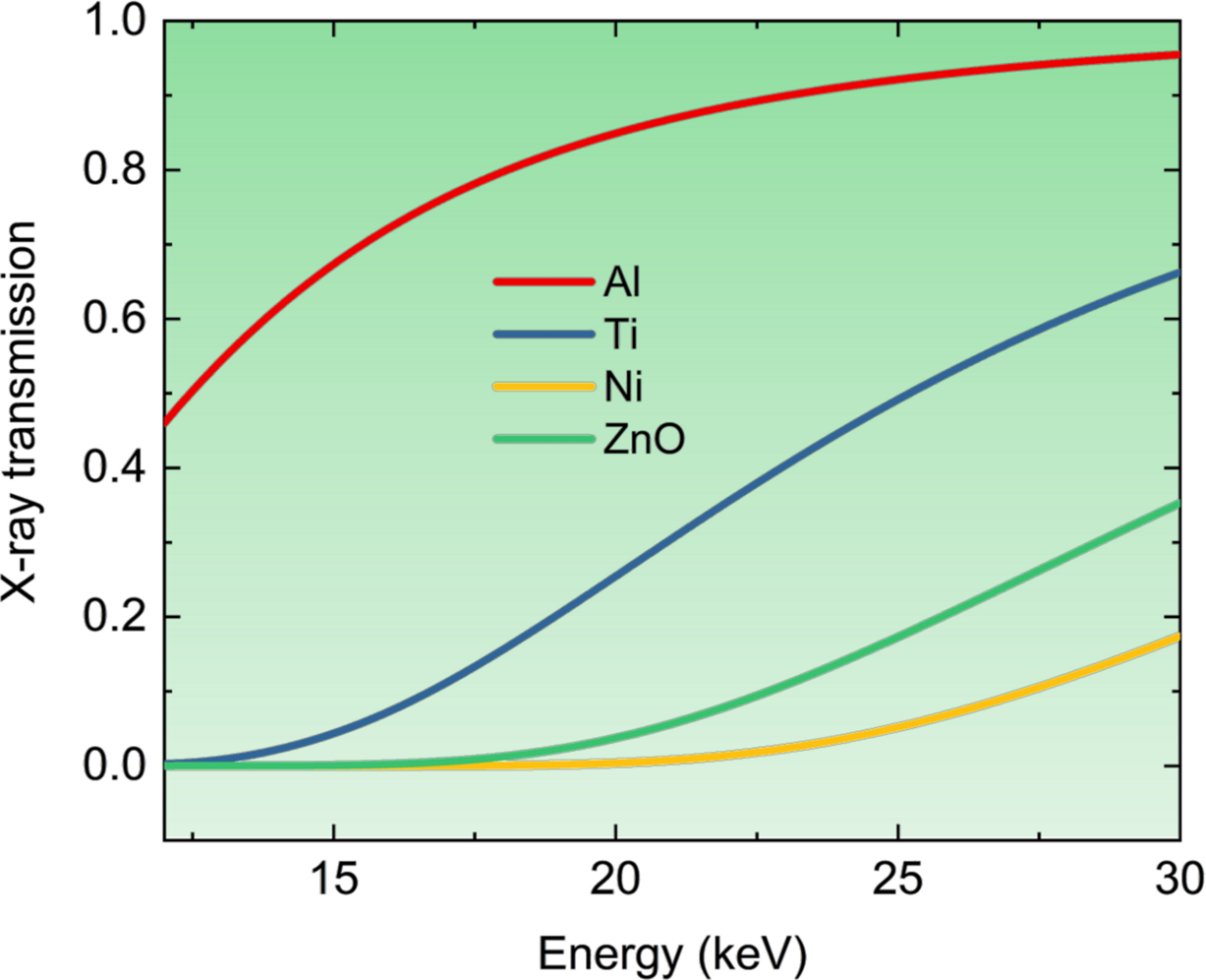
X-ray transmissions for various typical hard materials with different electron (mass) densities across an X-ray energy spectrum from 12 to 30 keV. The materials have a uniform thickness of 200 µm to ensure that the scattering data can reasonably represent bulk properties.

The second key point concerns the materials’ composition. The interaction between X-rays and matter hinges on the electron density of the material, as X-rays mainly interact with electrons. A material with higher electron density has a stronger interaction with X-rays; in general, materials become more opaque to X-rays as their electron density increases. Hard materials often contain heavier elements with more electrons, leading to increased photoelectric effect, Compton scattering and elastic scattering and a reduced transmission. Fig. 3 ▸ illustrates the X-ray transmission through several standard hard materials [three metals: aluminium (Al), titanium (Ti) and nickel (Ni); and one ceramic: zinc oxide (ZnO)], within the energy range 12–30 keV. These materials are assumed to be fully dense, with mass densities of 2.7 g cm^−3^ (Al), 4.5 g cm^−3^ (Ti), 8.9 g cm^−3^ (Ni) and 5.6 g cm^−3^ (ZnO), respectively. We also assume a material thickness of 200 µm. At this thickness, the impact of the surface structure deviation from the bulk on the measured data may be considered negligible for evaluation of the material’s bulk structure (Zhang, Stoudt *et al.*, 2021 ▸). The calculated transmission, based on these assumptions, clearly shows that, for most of these materials, an X-ray energy above 12 keV, and often above 20 keV, is necessary for significant transmission for X-ray scattering measurements in transmission geometry. For practical purposes, we set the minimum X-ray energy for studying hard materials at 12 keV, while noting that X-ray energies above 20 keV are likely to be needed for many types of hard materials.

For pinhole SAXS instruments, it is essential to understand that the minimum of the scattering vector magnitude *q*_min_ is significantly affected by the X-ray energy (wavelength), as the scattering angle 2θ = 2*a*sin(*q*λ/4π). To measure the same *q*_min_ with a wavelength that is half as long, measurements must be taken at angles approximately twice as small. For long pinhole devices, this requires doubling the sample-to-detector distance, which may not always be feasible. Additionally, achieving high resolution often relies on focusing the X-rays onto the detector plane. In this context, the performance of the focusing optics becomes crucial, and it is important to note that this performance is also dependent on the X-ray energy (Matsuda *et al.*, 2008 ▸; Bajt *et al.*, 2018 ▸). These strict requirements complicate the design of pinhole instruments intended to operate at energies greater than 20 keV while adhering to the USAXS criteria. In reality, most pinhole instruments capable of accessing the USAXS regime operate at energies below 8 keV. This energy limitation makes it challenging, if not impossible, to meaningfully measure electron-dense hard materials.

The Bonse–Hart USAXS instruments are less prone to such restrictions because the *q* resolution and *q*_min_ are defined by the Darwin width of the crystal optics. However, as the energy increases, the Darwin width, which is on the order of arcseconds, becomes narrower, making the stability and precision of the optics a major challenge. For example, during a measurement, the collimating crystals, which do not move, must be highly stable to ensure the incident X-ray beam on the sample does not change. The analyzer crystals, which rotate during the measurement, must maintain the same degree of stability as the collimating crystals, while also achieving rotations with precision comparable to the width of the Darwin curve. Meeting the stability and precision requirements is far from trivial and becomes more difficult as the operating energy increases. Hence, these requirements demand careful instrumental design and consistent improvement. This gradual improvement is, in fact, how the APS USAXS instrument has progressively increased its standard operating X-ray energy, starting from 10 keV in 1999, to 12 keV around 2002, 18 keV around 2008, 21 keV around 2015, and – for the APS-U instrument – 24 keV in 2023 (Ilavsky *et al.*, 2013 ▸, 2012 ▸). The current validated energy range for this instrument is 12–27 keV, with the upper limit determined by the capabilities of the available beamline (undulator and monochromator).

### 2.3. Key comparison between Bonse–Hart USAXS and pinhole USAXS

Although both pinhole camera and Bonse–Hart designs of USAXS instruments can conduct meaningful measurements on hard materials, significant differences exist that users should evaluate before designing an experiment. These distinctions might be obvious to instrument scientists who build these instruments, but they are not always considered or understood by users. For clarification, we will highlight several key aspects of these differences and compare the general performance of the instruments to assist users in deciding which to choose.

#### 2.3.1. Beam size (sample volume)

Beam size is often overlooked by users, yet it plays a crucial role in USAXS applications, especially when using pinhole cameras. On one hand, the beam size on the detector plane directly influences the *q* resolution (Δ*q*) in pinhole cameras. To achieve the high *q* resolution necessary for USAXS measurements (approximately 10^−4^ Å^−1^), the beam size on the detector plane must be smaller than, or at least comparable to, the pixel size of the detector. On the other hand, a smaller beam size on the sample is beneficial for enhancing spatial resolution, typically achieved by focusing the beam on the sample plane. However, this focus on the sample plane often results in a lower *q* resolution for pinhole USAXS devices, demanding a compromise to meet both needs.

Furthermore, for accurate measurement of scatterer dimensions, the beam size must be significantly larger than the scatterer size. This principle underpins the statistical validity of a small-angle scattering experiment (Guinier *et al.*, 1979 ▸). Ideally, for orientational averaging and to observe a representative sample volume, the volume illuminated by the X-ray beam should be several orders of magnitude larger than the volume of the scattering inhomogeneities being measured. Fulfilling this requirement with a beam size on the order of 2π/*q*_min_ represents a significant challenge for a pinhole camera.

Conversely, Bonse–Hart instruments do not suffer from this restriction because their Δ*q* is determined solely by the crystal optics used and is independent of the beam size. At the APS-USAXS instrument, for example, users routinely use a beam size of up to 1 × 1 mm along transverse directions. This feature is particularly beneficial for studying minerals, alloys and other samples that may contain substantial microscopic inhomogeneities.

Given the diverse beam size needs of different experiments, which depend heavily on the specific scientific question being addressed, and for the sake of simplicity, we will not delve into specific beam-size values. However, it is crucial for users to carefully assess whether an appropriate beam size is available on the instrument they intend to use and to be aware of the limitations associated with instruments that rely on a highly focused beam to access the USAXS regime.

#### 2.3.2. Anisotropy

Pinhole cameras provide several inherent advantages in measurements, one of which is the inclusion of a 2D detector. The 2D nature of the detection facilitates effective capture of scattering anisotropy, making these instruments highly useful for observing the structural anisotropy intrinsic to the materials or how materials respond to orientational stimuli, such as uniaxial tension or electric fields.

Bonse–Hart USAXS measurements, as shown in Fig. 2 ▸(*b*), collect scattering data using what is known as slit-smearing geometry. In this setup, the detector accepts the scattering intensity within the acceptance window of the crystal Darwin curve. This leads to very high angular (*q*) resolution in the direction of the reflection [for example, vertical in Fig. 2 ▸(*b*)]. However, in the direction perpendicular to the reflection, the acceptance window (slit length) is wide, resulting in slit-smearing (Ilavsky *et al.*, 2002 ▸). This slit-smearing makes it necessary to adopt a desmearing step to compute the differential scattering cross-section (Strobl, 1970 ▸). Typically, this desmearing step, employing common routines such as the Lake method (Lake, 1967 ▸), assumes isotropic scattering. This assumption fails for materials with highly anisotropic scattering profiles. It is, however, still possible to capture anisotropic scattering patterns in the Bonse–Hart configuration. This is achieved by introducing a second pair of collimating crystals before the sample and a second pair of analyzing crystals after the sample, with the diffraction plane of the second pair perpendicular to that of the first. This arrangement effectively creates pinhole collimation and detection (Ilavsky *et al.*, 2002 ▸). The 2D Collimated USAXS approach is less efficient in terms of X-ray usage and also poses greater challenges in alignment and operation. As such, it is reserved for cases where its application is deemed critically important, such as in the study of strongly scattering anisotropic ceramic samples where typical pinhole cameras are not an option (Kulkarni *et al.*, 2006 ▸).

#### 2.3.3. Time resolution

Understanding structural transformation kinetics of materials is at the core of modern materials science because it provides essential insights into the behavior of materials under various conditions. To capture such kinetics, measurements need to be time-resolved. Time resolution represents another major difference between pinhole cameras and Bonse–Hart USAXS measurements.

Modern pinhole cameras are equipped with area detectors capable of high-repetition-rate measurements (1 Hz and faster). With the ever-increasing brightness of synchrotron sources, flux densities on samples often exceed 10^13^ photons s^−1^mm^−2^. Consequently, the frame rate of area detectors has become the limiting factor in the speed of measurements (König *et al.*, 2023 ▸).

However, the repetition rate of the detector is just one aspect of the true time resolution of a measurement. Unlike ultra-small angle neutron scattering (USANS) instruments, which are typically standalone devices capturing a fixed range of *q* values (Agamalian *et al.*, 1998 ▸; Rehm *et al.*, 2013 ▸; Hainbuchner *et al.*, 2002 ▸) and whose data are often combined with SANS data from other instruments, USAXS instruments, whether pinhole or Bonse–Hart, aim to capture a wide *q* range using a single instrument to acquire structural information.

For the pinhole camera, the actual *q* range available to the area detector is constrained by its physical size, the distance between the sample and the detector, and the dynamic range of the detector. The physical size and the sample-to-detector distance, combined, define the detecting solid angle. Area detectors cover the measured solid angle with pixels spaced linearly, and each pixel covers a fraction of the measured solid angle. This fraction represents the fundamental *q* resolution of each pixel. Given that most detectors today have pixel dimensions on the order of 1000 × 1000, this *q* resolution is approximately 1/1000 of the full *q* range covered by the area detector. If we assume the *q* resolution meets the requirement of a USAXS instrument (≃10^−4^ Å^−1^), a single area detector with 1000 pixels along each transverse direction will cover a *q* range of over three orders of magnitude (10^−4^ to 10^−1^ Å^−1^). However, for small-angle scattering, we also must consider the scattering intensity. Following Porod’s law, where the intensity falls off as *q*^−4^, a *q* range over three orders of magnitude leads to an intensity range that covers 12 orders of magnitude. Even the best detector today can only measure over a dynamic range of 10^7^ photons pixel^−1^s^−1^. This insufficient dynamic range becomes a limiting factor for the *q* range a single area detector can cover in SAXS devices.

There are multiple mitigation strategies. The most common approach is to have the detector on a rail, perform multiple SAXS measurements at different sample-to-detector distances and merge the resulting SAXS data together. This configuration diminishes the fast detection empowered by area detectors, making USAXS measurements over a broad *q* range a slow process. An alternative method is to use multiple detectors positioned at different distances, as in the case of DNDCAT at the APS (Weigand & Keane, 2011 ▸). However, such a setup is often optimized for X-rays of a specific wavelength, and complete coverage of a continuous *q* range must be optimized carefully. We also emphasize that such a multi-detector setup provides the best compromise between a broad *q* range and high-frequency (>0.1 Hz) measurements.

Bonse–Hart USAXS employs a point-scanning technique, as opposed to collecting scattering data over a range of *q* simultaneously in a pinhole configuration. This approach makes USAXS instruments significantly slower, when compared with a multi-detector setup, with data acquisition time depending on X-ray flux, the scanned angular range and the number of data points collected. We note that data collection can be sped up by utilizing fly scanning (Ilavsky *et al.*, 2018 ▸), where data are collected while continuously moving the crystals and detector, without stopping at each angular point. This method can reduce the data collection time by a factor of 2–5× in some cases, making data acquisition from 1 × 10^−4^ to 0.3 Å^−1^ possible in under 30 s. Such a performance is comparable to, or exceeds, a pinhole camera where the detector is mounted on a rail system.

#### 2.3.4. Absolute intensity calibration

Absolute intensity calibration is an often overlooked but critical element of SAXS measurement and analysis. This calibration allows for the quantitative analysis of sample characteristics such as the number/volume size distribution of scattering objects. It also enables the direct comparison of SAXS data across different experiments and instruments, ensuring the replicability and standardization of measurements.

The absolute standard calibration for a Bonse–Hart instrument is an inherent feature of the instrument because no beam stop is used, and the analyzer crystals scan through the incident beam to capture the forward scattering (*q* = 0) intensity (Long *et al.*, 1991 ▸). Such primary calibration is the basis of an *ad hoc* SAXS intensity standard (Zhang *et al.*, 2010 ▸) and a standard reference material certified and issued by the National Institute of Standards and Technology, USA [NIST Standard 3600 (Allen *et al.*, 2017 ▸)], both using glassy carbon. These standards are now used in hundreds of laboratories worldwide.

Pinhole cameras often include a beam stop, which complicates the accurate measurement of forward scattering intensity. Consequently, to achieve absolute intensity calibration, a secondary standard is needed, like the NIST standard from a Bonse–Hart instrument. Although this represents an additional step, we strongly advocate making absolute intensity calibration a necessary component of every SAXS, and USAXS, measurement.

## 3. USAXS instruments worldwide

As recently as two decades ago, only a few instruments worldwide could access the USAXS regime. However, the exciting scientific possibilities and the potential for *in situ* studies have led to the development of many instruments in recent years. These instruments are capable of accessing a broad range of scattering vectors, significantly contributing to our understanding of materials science.

Many of these devices are pinhole instruments, often exceeding 8–10 m in length and generally operating with X-ray energies below 8 keV. With optimized beam stops, focusing, detectors and strategies for reducing parasitic scattering, these instruments can satisfactorily meet the USAXS criteria. Their existence has greatly enhanced our understanding of soft materials, such as polymers, colloids and biological materials. However, achieving the USAXS *q*-range at the high X-ray energies (>20 keV) required for studying hard materials is only feasible for pinhole devices when the sample-to-detector distance is longer than approximately 20 m. Such extensive equipment in a synchrotron facility can be cost-prohibitive, including the construction of long hutches and the procurement of lengthy flight tubes, which alone can cost millions of USD. Such long instruments may also suffer from relatively poor time resolution, especially if a single detector is used to acquire data over a broad *q* range.

**Figure 4:**
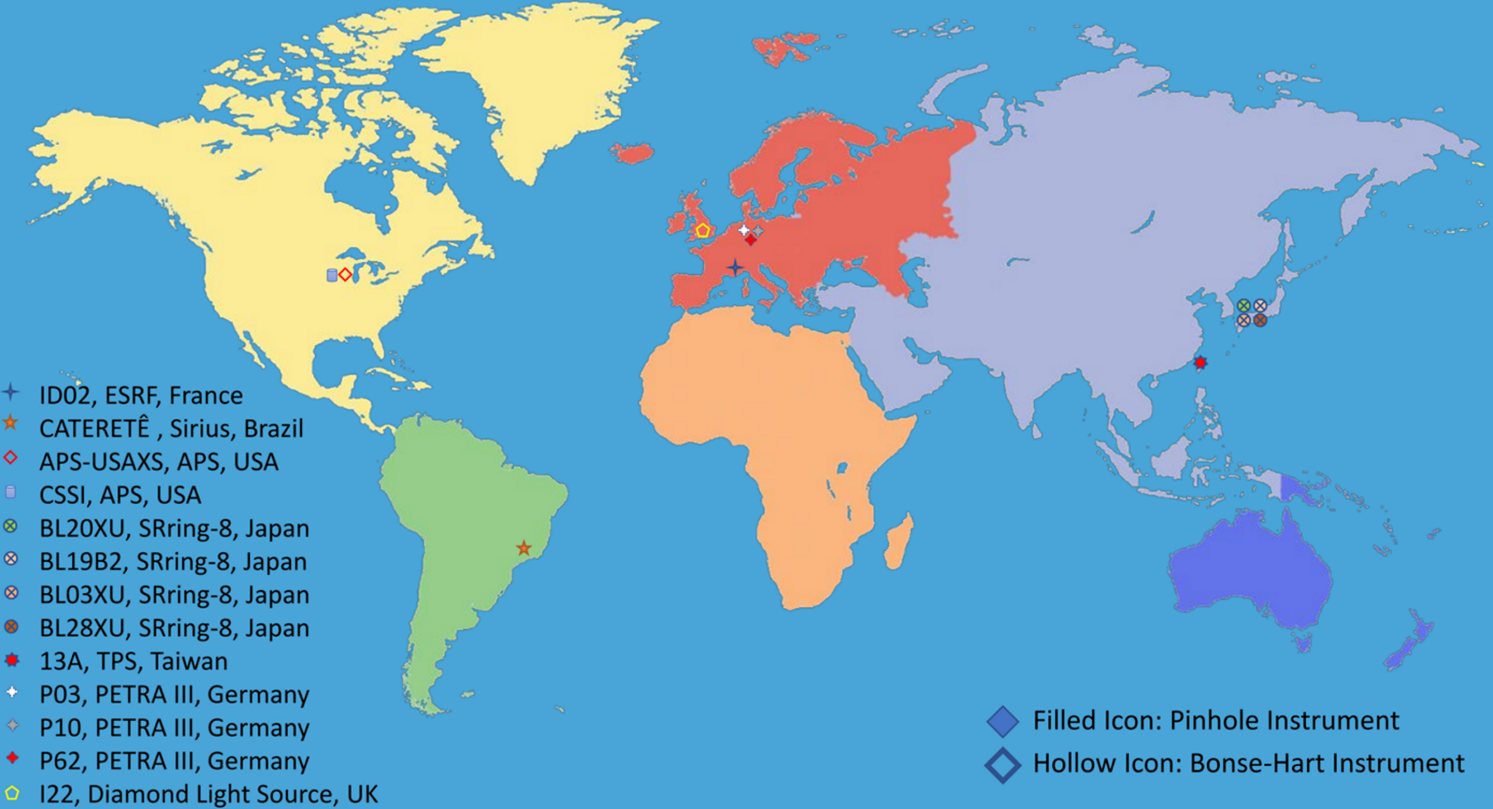
Geographical distribution of synchrotron based USAXS instruments available worldwide. Filled icons indicate pinhole instruments and hollow icons denote Bonse–Hart instruments. Note that the geographical locations are approximate.

Today, most synchrotrons offer one or more SAXS instruments, with many claiming to provide USAXS capabilities, at least at low X-ray energies. These instruments each have unique characteristics, influenced by specific design choices, source capabilities and the needs of their respective local communities. We will succinctly describe these instruments in this section. They are highlighted on a world map (Fig. 4 ▸), enabling users globally to identify the instruments that are most geographically convenient for them. While most are well suited for research on soft matter, we advise users to exercise caution and refer to the criteria established in earlier sessions when considering measurements for hard materials. We also emphasize that these capabilities are continually evolving, as beamlines are upgraded with new detectors or X-ray optics, rendering this summary a snapshot of the current state. Note that SAXS instruments not claiming USAXS capabilities have been excluded from this review, and it is possible that some instruments may have been inadvertently overlooked by the authors.

### 3.1. Fourth-generation sources

The fourth-generation synchrotron source, utilizing multi-bend achromat technology, represents a significant leap forward in synchrotron based X-ray science. These sources dramatically reduce the emittance of the electron beam, which in turn drastically increases the brightness and coherence of the X-ray beams. This leads to significantly improved performance for applications such as X-ray photon correlation spectroscopy (XPCS) (Sandy *et al.*, 2018 ▸) and coherent X-ray diffraction imaging (CXDI) (Chapman & Nugent, 2010 ▸) in the USAXS regime. Consequently, we have listed the USAXS instruments at fourth-generation sources separately by facility, while noting that many current facilities have plans to upgrade to a fourth-generation source. As a result, this list is expected to grow over time.

### 3.2. European Synchrotron Radiation Facility, Grenoble, France

Beamline ID02 at the European Synchrotron Radiation Facility (ESRF) (Narayanan *et al.*, 2022 ▸, 2023 ▸) is an outstanding example of a synchrotron based pinhole USAXS device, capable of achieving the *q* range <0.0001–6 Å^−1^ for suitable samples by tuning the X-ray energy, owing to its 34 m long detector chamber. At 12.2 keV, the *q* range 0.0001––5 Å^−1^ is attainable with two sample-to-detector distances and a single setting for beam collimation/focusing. The beamline has a coherent flux of 10^12^ photons s^−1^mm^−2^ at 12 keV and supports materials dynamic studies within the energy range 8–20 keV at a high time resolution of up to 23 kHz (for XPCS), which will soon be upgraded to 56 kHz with a Rigaku detector (again for XPCS).

### 3.3. Sirius – the Brazilian Synchrotron Light Laboratory, Campinas, Brazil

The CATERETÊ beamline at Sirius (Meneau *et al.*, 2021 ▸) features a 28 m vacuum chamber hosting the Medipix (3k × 3k pixels^2^) in-vacuum detector, allowing time-resolved SAXS, USAXS, XPCS and CXDI measurements. The beamline operates between 3 and 24 keV. The minimum *q* value is 0.000075 Å^−1^ at 6 keV and 0.0004 Å^−1^ at 17 keV.

### 3.4. Advanced Photon Source, Argonne, United States

APS, the largest high-energy synchrotron radiation facility in the United States, is currently undergoing an upgrade to the APS-U as this review is written. As part of this upgrade, the APS-U is installing multiple new pinhole based instruments to offer USAXS capabilities, in addition to the current, Bonse–Hart APS-USAXS instrument. These pinhole devices, including the ‘Grand Tube’ of the Coherent Surface Scattering Imaging (CSSI) beamline, featuring SAXS chambers ranging from 10 to 22 m and high coherence beams within 5–20 keV energy ranges, will support USAXS/SAXS/WAXS techniques in both transmission and grazing incidence modes, along with coherence techniques like XPCS, CDI and ptychography. Operations are expected to begin in late 2024, with final capabilities to be fully determined at that time.

### 3.5. Prior-generation sources

Prior-generation synchrotron sources, especially third-generation sources, provide X-ray flux similar to fourth-generation sources but lack the high coherence, particularly at the high X-ray energies of interest for hard materials research. Devices at these facilities can match the capability of fourth-generation instruments for SAXS/USAXS measurements, but coherence based techniques are less powerful.

### 3.6. Super Photon ring-8 GeV, Hyogo, Japan

Super Photon ring-8 GeV (SPring-8) has several USAXS/SAXS instruments. Most notably, BL20XU, with a detector distance of 160 m, is likely to be the longest pinhole based USAXS instrument worldwide (Yagi & Inoue, 2003 ▸). With 8 keV X-rays, it can access *q*_min_ = 2.5 × 10^−5^ Å^−1^, which outperforms the currently best *q*_min_ of a Bonse–Hart instrument [3 × 10^−5^ Å^−1^ using an Si(440) optics at 24 keV]. This instrument regularly operates at 23 keV but has a limited *q* range defined by the size of the beam stop (10 mm in diameter) and the inner diameter of the vacuum duct (80 mm), and requires SAXS data to be collected with other devices at SPring-8, such as BL40XU and BL40B2 to supplement the USAXS data for a broader *q* range.

BL19B2, a bending-magnet beamline, offers a 0.7–3 m SAXS and a 42 m USAXS with the energy range 18–30 keV, covering a broad *q* range, allowing USAXS measurements at 18 keV (Osaka *et al.*, 2016 ▸). These techniques cannot be combined in one experiment simultaneously.

BL03XU, a dedicated soft material research beamline (Masunaga *et al.*, 2011 ▸), can perform USAXS measurements, in addition to SAXS, XRD and grazing incident measurements. At 12.4 keV, it can achieve a *q* resolution of 2 × 10^−4^ Å^−1^.

Finally, BL28XU is a pinhole instrument designed to support anomalous USAXS measurements (Nakanishi *et al.*, 2024 ▸). With time-resolved capability to perform measurements at 17 energies near an absorption edge within 30 s, and a *q*_min_ near 0.0005 Å^−1^, it opens new possibilities to exploit element-specific studies in complex materials.

### 3.7. Taiwan Photon Source, Hsinchu, Taiwan

Beamline 13A, the BioSAXS instrument at the Taiwan Photon Source (TPS), operates between 4 and 23 keV (Shih *et al.*, 2022 ▸). With a 12 m vacuum chamber, the instrument can access a broad *q* range between 0.0003 and 4 Å^−1^.

### 3.8. Positron–Electron Tandem Ring Accelerator, Hamburg, Germany

The Positron–Electron Tandem Ring Accelerator (PETRA III) is one of the world’s brightest storage-ring based high-energy synchrotron X-ray research facilities. It has several beamlines that deliver USAXS capabilities. For example, beamline P03 provides USAXS measurement capability with a moderately focused beam and operates between 7 and 21 keV (Buffet *et al.*, 2012 ▸). Beamline P10, a coherent scattering beamline, offers (coherent) USAXS capabilities with a 21.3 m sample-to-detector distance (Vartanyants *et al.*, 2020 ▸). The maximum accessible *q* value is limited to ≃0.02 Å^−1^ at 8 keV. Since no focusing is possible for this setup, it operates at low flux density limits, beneficial to a broad range of soft materials susceptible to radiation damage.

We note that beamline P62, the SAXSMAT beamline, is designed to perform simultaneous (U)SAXS and WAXS measurements (Haas *et al.*, 2023 ▸). At this beamline, the X-ray energy can be continuously tuned from 3.5 to 35 keV using an Si(111) double crystal monochromator. Its primary focus includes anomalous X-ray scattering techniques, SAXS/WAXS tensor tomography, and *in situ* and *operando* studies. Typically, the sample geometry is set up in transmission mode. The (U)SAXS detector used is an Eiger2 X 9M from Dectris, whereas the WAXS detector is an Eiger2 X 4M DESY, also from Dectris.

### 3.9. Diamond Light Source, Oxfordshire, UK

Last but not least, a collaboration between Diamond Light Source (DLS), UK, and the Federal Institute for Materials Research and Testing (BAM), Germany, has recently created a low-cost Bonse–Hart USAXS module as an add-on to the pinhole instrument at I22 of the DLS (Pauw *et al.*, 2021 ▸). This plug-in module makes use of Si(220) crystal optics and extends the *q*_min_ of I22 from 0.002 to 0.00015 Å^−1^. This over one order of magnitude increase in the *q* range could be realized under 30k Euro (in 2021), thus creating a cost-effective pathway for many other SAXS instruments worldwide to add the USAXS capability.

The construction of synchrotron instruments requires long-term planning. Despite this, many of the instruments mentioned previously have been brought online in the last decade, and others, such as P62 at PETRA III, are planned to include measurement capabilities in the USAXS regime. We are optimistic that with continued technical development and planning, the USAXS capabilities worldwide will keep expanding to pave the way for new discoveries and technological innovations in various fields.

## 4. USAXS science examples for hard materials

With decades of dedicated development from groups of scientists worldwide, and recent advancements in synchrotron instrumentation (including detector development) enabling the beam stability, focusing conditions and detection efficiency required for successful USAXS measurements in the hard X-ray regime, USAXS has emerged as a pivotal analytical technique in the investigation of hard materials. It offers insights into their microstructural characteristics; hierarchical organization; and, through *in situ* experiments, microstructural evolutions under external stimuli. The application of USAXS impacts a diverse range of hard materials, including alloys, ceramics, geological specimens and advanced technological materials. These studies have significantly advanced our understanding of their internal structures at the nanometre to micrometre scales, providing the necessary fundamental structural insights to modern materials science and driving innovations in materials design and development as well as process optimization. In this section, we cover selected material classes. In each class, we provide a brief overview highlighting the significance of USAXS measurements to these materials and use examples to illustrate these points. Note that we will not make distinctions between data acquired using pinhole USAXS and Bonse–Hart USAXS because the instrumentation serves only one goal, allowing data in the necessary *q* range to be properly captured.

### 4.1. Alloys

Alloys are among the most widely used structural materials and carry enormous economic value and impact. Designed to support and resist loads, alloys have a broad range of applications, from as mundane as shelves to specialty alloys targeted as advanced aerospace materials. Without exception, alloys possess hierarchical structures from the atomic scale to the macroscopic scale, which play a critical role in defining their properties and performance in these applications. For example, at the nanoscale, the atomic arrangement and distribution of phases, grain boundaries and defects govern mechanical properties such as strength and hardness. At larger scales, properties such as fatigue and wear resistance can be tailored by manipulating microstructural features. Such multiscale structural architecture requires careful design to ensure the alloys can meet demanding requirements in the most high-valued applications of these materials, such as in high temperature, high stress and corrosive environments.

USAXS and its complementary techniques are uniquely positioned to probe the physical properties of alloys across a wide range of length scales, from nanometres to micrometres. When high-energy X-rays are utilized, this capability allows for bulk, quantitative and statistically meaningful characterization of structural and morphological features within the alloys, such as grain size and distribution (Mori *et al.*, 2018 ▸); precipitate formation and its size distribution (Zhang, Ilavsky *et al.*, 2021 ▸; Zhang *et al.*, 2016 ▸); porosity (Guo *et al.*, 2022 ▸; Hammons *et al.*, 2022 ▸); localized compositional variation (Imhoff *et al.*, 2012 ▸); interfacial roughness (Keist *et al.*, 2020 ▸); and under challenging measurement conditions, structure of dislocation (Long *et al.*, 2000 ▸; Long & Levine, 2005 ▸).

Among these, the characterization of precipitate structure and formation kinetics holds a special place and represents the most prominent and widely used application of USAXS for alloys. SAXS, as a matter of fact, is among the most powerful tools available for characterizing precipitates. Several review papers have nicely described the scientific case and the significance of the results acquired from SAXS (De Geuser & Deschamps, 2012 ▸; Deschamps & Hutchinson, 2021 ▸). In contrast to SAXS, which provides a more limited *q* range, USAXS has the added advantage of capturing scattering intensity at the larger size range. This is important because the quantitative characterization of the scattering from precipitates tens of nanometres in size still requires an accurate determination of the scattering background and, in alloys, the grains, with the grain boundary typically having different scattering length density than the grain interior, scatter. The grain sizes are typically on the length scale of micrometres, making their Guinier region unavailable to SAXS or even USAXS. However, the Porod region of the grain scattering remains and interferes with modeling and interpretation of the scattering from smaller precipitates. The expanded *q* range also allows multiple species of precipitates to be distinguished and analyzed simultaneously (Jia *et al.*, 2020 ▸). This capability enhances our understanding of the complex interactions and transformations within alloys, allowing for a more comprehensive analysis of their microstructural evolution.

**Figure 5:**
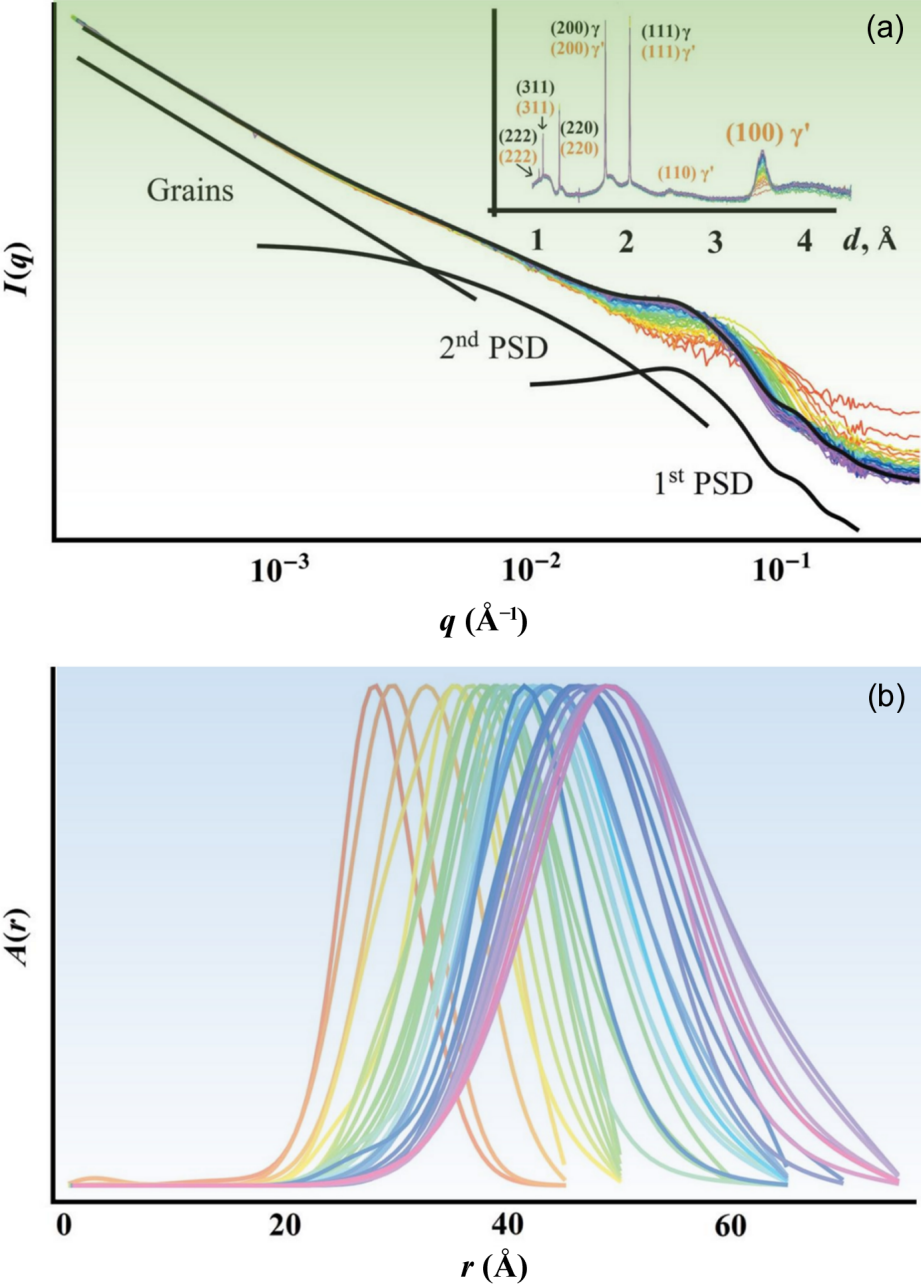
USAXS measurements reveal the time-dependent size distribution of the γ′ precipitates in a model ternary Ni-based alloy. (*a*) USAXS data allow for accurate characterization of the contribution of scattering intensity from the grain structure, a time-invariant second size distribution (second PSD) and the dynamic size distribution attributable to the γ′ precipitates (first PSD). (*b*) Time-dependent size distribution acquired using a novel Bayesian–MaxEnt analysis. This figure was adapted from Andrews *et al.* (2017 ▸).

The true power of USAXS, or SAXS in general, in analyzing precipitate formation in alloys, lies in its ability to probe the precipitation kinetics *in situ*. In these experiments, the X-ray beam monitors the same sample volume, making the transformation kinetics readily available upon proper analysis. In particular, it allows direct observation of coherent precipitates, which can be difficult to resolve using other *in situ* techniques such as X-ray diffraction. Here, the compositional difference provides the necessary contrast for SAXS. In a recent example (Fig. 5 ▸), Andrews *et al.* (2017 ▸) leveraged the broad *q* range of USAXS to analyze the nucleation, growth and coarsening of γ′ precipitates in a model Ni-based alloy during heat-treatment processes. The USAXS analysis provided insights into the early stages of precipitate evolution, which are critical for understanding the strengthening mechanisms in these alloys. Moreover, this study developed a Bayesian–MaxEnt (Maximum Entropy) analysis technique to overcome challenges posed by low-*q* scattering and structure factor effects, demonstrating their effectiveness in analyzing precipitate size evolution.

We note that USAXS has also contributed to our understanding of the microstructural heterogeneities introduced by the additive manufacturing (AM) process of metals, a recent focus area in metallurgy, or materials science in general. AM processing differs from traditional processing due to its rapid cooling rates and localized heating, resulting in significant spatial variations in microstructures and a drastically different response of AM metals to standard heat treatments compared with their cast or wrought counterparts (Campbell *et al.*, 2020 ▸). For example, USAXS results have elucidated the formation mechanism of the detrimental δ phase precipitates in AM Inconel 625, an Ni-based superalloy (Zhang, Levine *et al.*, 2018 ▸). This phase, typically forming after thousands of hours at temperatures higher than 800°C, appears in substantial volumes within 1 h at 800°C. Using USAXS data, time–temperature–transformation (TTT) curves for the δ phase were constructed (Lindwall *et al.*, 2019 ▸), and a general methodology for investigating the response of AM materials to heat treatments was established. The USAXS data are also integral components of the Additive Manufacturing Benchmark Series (AM-Bench) of 2018 (Zhang *et al.*, 2019 ▸) and 2022 (Zhang *et al.*, 2024 ▸), aimed at using high-pedigree experimental data to guide the development of computer models to ensure the continued development of AM technologies.

### 4.2. Ceramics

Traditionally used as structural materials, ceramics have become an essential class of material in modern science, technology and industry development because of their unique properties and applications (Allen, 2005 ▸, 2023 ▸). Ceramics exhibit exceptional high-temperature stability, high hardness and wear resistance, wide-ranging electrical properties from insulators to semiconductors to superconductors, and high chemical stability, making them the materials of choice for numerous applications such as energy storage and conversion, aerospace components, and electronic devices.

**Figure 6:**
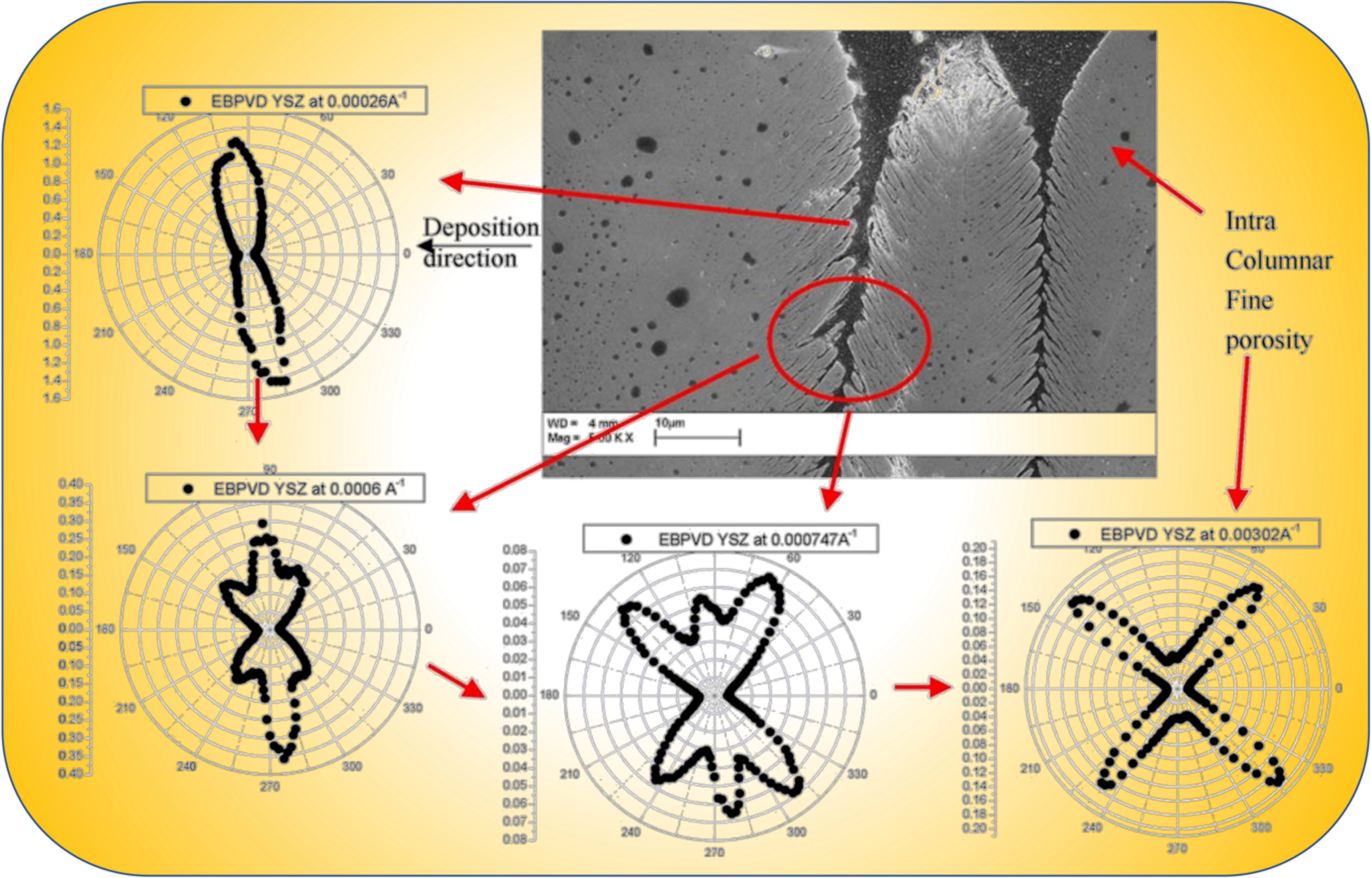
2D USAXS measurements revealing the size (*q*) dependent microstructural anisotropy in a Y_2_O_3_-stabilized ZrO_2_ coating produced by electron beam-physical vapor deposition. This figure was adapted from Kulkarni *et al.* (2006 ▸).

One of the significant applications of USAXS in the study of ceramic materials involves analyzing the microstructures of thermal barrier coatings (Renteria *et al.*, 2007 ▸; Kulkarni *et al.*, 2004 ▸; Ilavsky, 2010 ▸). These coatings, which can be created using various technologies such as electron beam physical vapor deposition (EB-PVD) and different types of thermal spraying, result in distinct microstructures. Applied to engine components, these coatings act as a thermal insulation layer, protecting the critical components from the extreme temperatures generated during engine operation. This allows engines to operate at higher combustion temperatures, enhancing efficiency and performance. A key feature for these coatings to function effectively as thermal barriers is a high level of porosity, as this characteristic significantly reduces thermal conductivity. The pore structure is complex. For example (Fig. 6 ▸), in a study of Y_2_O_3_-stabilized ZrO_2_ coating prepared by EB-PVD (Kulkarni *et al.*, 2006 ▸), USAXS data revealed that the coating exhibits a hierarchical microstructure consisting of pores of at least three different sizes, consistent with observations made using SEM. The *q*-dependent anisotropic scattering behavior illustrates the volume-averaged hierarchy of pore sizes within the coating: intercolumnar pores with mean dimensions between 630 and 820 nm, intracolumnar feathery cracks between 100 and 200 nm, and nanometre globular pores ranging from 8 to 20 nm in diameter. This hierarchy is broadly consistent with the microstructures observed in SEM analyses, emphasizing the capability of USAXS to measure and quantify void sizes up to approximately 1.5 µm in diameter. This level of detailed, quantitative information is difficult to acquire using other techniques and is useful in optimizing the performance of these coatings for critical engine applications.

The sintering step is universal for ceramics processing. Interrogation of the pore size distribution during and after sintering is crucial for understanding and optimizing the properties and functionalities of ceramic materials. Sintering of ceramics typically occurs near the melting point of the ceramic materials, above 1200°C, making *in situ* investigation difficult. Typical advanced characterization techniques such as optical microscopy, SEM and TEM are destructive in nature, making detecting the evolution of pore size distribution throughout sintering a tedious, if not impossible, task. USAXS, and SANS, including USANS, are ideally positioned to provide nondestructive and *in situ* evaluations of the pore size for the high-electron-density ceramic materials. These data are beneficial for identifying the optimal sintering conditions, such as temperature and duration, to ensure a balance between grain growth and porosity reduction is achieved for targeted applications.

Though characterization of pore evolution of sintering, irrespective of sintering methods, presents a prime opportunity for USAXS to make a significant impact in ceramics engineering, this capability has been underutilized for several reasons. First, from an instrumental perspective, ceramic materials are electron-dense, requiring the use of high-energy X-rays. Secondly, the high-temperature furnace needed to reach typical sintering temperatures can be bulky and challenging to integrate into a pinhole-based USAXS instrument with a limited open-air path to reduce air scattering. Commercial heaters, such as Linkam, can reach up to 1500°C, but these temperatures may not suffice for a broad range of ceramics, especially refractory ceramics. Thirdly, process optimization has traditionally relied on a trial-and-error approach using in-house equipment. The lack of awareness of dedicated USAXS instruments, especially those capable of performing *in situ* studies, is a barrier. However, with the rapid development of computational platforms like Integrated Computational Materials Engineering (ICME), there is a growing need for *in situ* data to benchmark model predictions. The validated models can then provide reliable predictions for the sintering behavior of ceramic materials.

Finally, we want to bring attention to some exciting ceramics processing possibilities. Ceramic processing is energy-intensive, with sintering accounting for approximately 80% of the total energy consumption. Cold sintering (Guo *et al.*, 2019 ▸), a new ceramic processing technology capable of producing fully sintered material at temperatures below 200°C under a moderate uniaxial pressure (200 MPa), represents a new direction for making the ceramic industry more environmentally friendly. Using a dedicated sintering setup, *in situ* experiments of cold sintering of monopotassium phosphate (Allen *et al.*, 2021 ▸, 2022 ▸) and zinc oxide (Zhang *et al.*, 2023 ▸) have been performed, revealing the complex pore and interfacial evolution during the sintering process. These examples again highlight the need and significance of sample environment development to enable USAXS measurements to generate more impact in the advancement of ceramic engineering.

### 4.3. Geological materials

Geological materials represent another significant class of materials where USAXS has found many applications. Geological materials, including minerals, rocks and composites, exhibit structural organization on multiple scales, from the nano and microscale up to the macroscale. For example, clay minerals have layered silicate structures, carbonate rocks like limestone contain structured shell fragments and skeletal remains, and porous volcanic rocks feature a network of voids and channels. The hierarchical organization can significantly influence the properties of materials.

**Figure 7:**
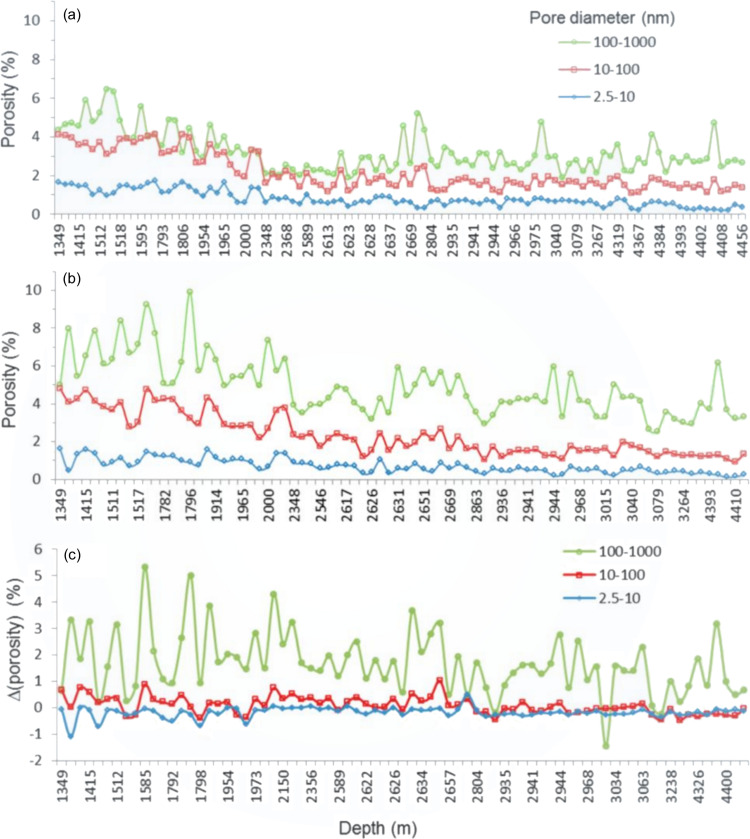
The pore structure of three types of pores from Silurian black shales from the Baltic Basin, Poland, across a wide range of depths along a burial diagenetic sequence. (*a*) Pore characteristics of the shale rocks in their air-dried state. (*b*) Pore characteristics in the dehydration state after drying at 200°C. (*c*) Difference in pore volume between the air-dried and dehydrated states, demonstrating the structural changes induced by dehydration. This figure was adapted from Lee *et al.* (2014 ▸).

Many current interests in geological minerals are driven by energy recovery, material extraction and climate needs (Gadikota *et al.*, 2017*b* ▸,*a* ▸; Zheng *et al.*, 2023 ▸). In pursuit of domestic energy security, many countries have turned to hydraulic fracturing (fracking), where high-pressured fluid is injected into the rock formation deep underground to extract hydro­carbons (in the form of oil and natural gasses). For successful extraction, the detailed pore structure of shale rock, the resource rock for oil and gas, must be understood in terms of pore sizes, pore distributions and the connectivities of the pores within the rock matrix (Anovitz & Cole, 2018 ▸). This type of knowledge is critical for predicting the flow behavior of the liquid through the rock and to optimize the fracking extraction efficiency. Fig. 7 ▸ presents an example from a comprehensive study (Lee *et al.*, 2014 ▸), where shale rock samples were systematically extracted from the Silurian black shales of the Baltic Basin, Poland, across depths ranging from 1416 to 4456 m. USAXS was primarily employed to investigate the pore structure, including pore volume and size distribution, in relation to depth and dehydration condition. Samples were analyzed in both their air-dried state, equilibrated at approximately 50% relative humidity, and prior to dehydration by drying at 200°C, facilitating a thorough comparison of the changes in pore structure induced by dehydration. The porosity increased with sample dehydration, in addition to the expected decrease with depth as per models of porosity evolution during burial. USAXS data strongly suggested that dehydration led to a notable increase in porosity, mainly due to the expansion of pore volumes ranging from 100 to 1000 nm in diameter. The exceptional agreement between USAXS results and immersion porosity methods underscores their significant contribution to shale research – USAXS not only provides precise measurements of total porosity, but also offers detailed insights into the distribution of pore sizes.

CO_2_ capture and storage (CCS) represents another area of significant current interest (Gadikota, 2018 ▸). During the solid-decomposition reactions of carbonates, understanding the evolution of internal porosity is critical. USAXS has been used to provide insights into the microstructural evolution of nanoscale pores, while accompanying WAXS measurements offer details about structural phase transformations. Multiple studies have investigated such transformations in a range of carbonate materials, from calcium carbonate (Strumendo *et al.*, 2022 ▸) and magnesium carbonate (Liu *et al.*, 2020 ▸) to other carbonate forms (Weber *et al.*, 2023 ▸; Gadikota, 2017 ▸), serving to optimize the CO_2_-sequestration reaction conditions and create more sustainable and effective CCS technologies.

Finally, we want to comment on the significance and potential opportunity of combining USAXS and USANS/SANS analysis in the investigation of geological materials. We group USANS and SANS together because USAXS offers a comparable *q*-range to that of USANS and SANS combined. With pore structure representing the overwhelming interest in the investigation of geological materials using small angle scattering approaches, USAXS and USANS/SANS provide different aspects of the same puzzle. X-ray scattering is highly sensitive to electron density differences, making it best suited to evaluate the structure and distribution of the inorganic components within the materials. Neutron scattering, on the other hand, is sensitive to isotopic contrast, making it suitable to distinguish the organic compounds residing inside the porous spaces. Combining these measurements allows organic and inorganic phases present in the materials to be evaluated together, which is essential for understanding geochemical processes such as hydro­carbon migrations and CO_2_ sequestration. We note that the best approach to realize such combined measurements might be to develop a dedicated instrument at a neutron facility, where desktop X-ray equipment provides simultaneous, supplementary X-ray scattering measurements to a SANS instrument. With the recent significant increase in the brightness of desktop X-ray sources, this combination has the possibility to provide similar time resolution between X-ray and neutron measurements. This integrated approach would enable a comprehensive analysis of geological samples, allowing the distribution and characteristics of both organic and inorganic matters be interrogated at the same time and offering unprecedented insights into their complex internal structures.

### 4.4. Other technological materials

In addition to the above mentioned classes of materials, USAXS has also provided critical data for evaluating the microstructures of a broad spectrum of other technologically significant materials. These include advanced cements (Maruyama *et al.*, 2017 ▸; Kupwade-Patil *et al.*, 2018 ▸; Allen & Livingston, 1998 ▸), glassy materials (Cai *et al.*, 2020 ▸; Paul *et al.*, 2019 ▸; Walter *et al.*, 1997 ▸), inorganic gels (Shoaib *et al.*, 2023 ▸; Jitianu *et al.*, 2017 ▸), energetic materials (Stepanov *et al.*, 2013 ▸; Dresselhaus-Cooper *et al.*, 2020 ▸), dental materials (Zhang *et al.*, 2014 ▸, 2012 ▸), solid oxide fuel cells (Witt *et al.*, 2020 ▸; Wang *et al.*, 2019 ▸) and many others. Although the range of materials is diverse, USAXS’s footprint remains limited compared with more common techniques such as X-ray diffraction and electron microscopy. One major factor contributing to this limitation is that small-angle scattering is traditionally viewed as a tool for soft matter research. Scientists and engineers in the field of hard materials are often not familiar with the capabilities of SAS in general, and USAXS in particular. Therefore, it demands continued outreach by experts to broaden the user community, ensuring that the structural insights USAXS can offer are better utilized to create a more significant impact.

## 5. Opportunities and challenges

As this review is written, the synchrotron X-ray landscape is truly transforming. The maturation of multi-bend achromat storage rings has overcome limits on synchrotron X-ray diffraction in the hard X-ray regime. The high brightness and coherence of the fourth-generation synchrotron enable rapid development of coherent scattering techniques, allowing access to a temporospatial domain never available before. Machine learning has emerged as an essential component of modern society, with large language models such as *ChatGPT* and *Gemini* serving as some of the most visible and successful examples. With the developments happening at synchrotron sources worldwide geared toward establishing more measurement capabilities in the USAXS regime, this is genuinely a time of opportunities for USAXS, and realizing these opportunities will require overcoming many challenges. Here, we will list a few primary opportunities for hard materials that we identify, within our knowledge and limitations.

### 5.1. Multimodal measurements

USAXS is rarely used as a standalone technique to understand the structural hierarchies in natural and engineered materials. It is common for USAXS/SAXS/WAXS data to be acquired on the same sample, ideally within the same sample volume, to provide nondestructive and statistically meaningful structural information over a broad range of length scales, either *in situ* or ex situ. This combined measurement capability should always be a component of a USAXS instrument, whether it is a pinhole instrument or a Bonse-Hart instrument. While realizing this capability for Bonse–Hart instruments require sequential measurements, these measurements can be achieved simultaneously for pinhole instruments. However, the long-range motion of the detector on the rail to capture scattering data across a continuous *q* range should be avoided, as it diminishes the value of time-resolved experiments, one of the most powerful and unique capabilities that synchrotron pinhole instruments provide. We recommend careful consideration of detector configurations during the planning phase of new instruments or instrument upgrades. A multi-detector configuration, such as the one at the DNDCAT of the APS (Weigand & Keane, 2011 ▸), should always be considered.

USAXS probes scattering homogeneities on scales from tens of nanometres to micrometres, allowing it to seamlessly connect to the typical spatial resolution that can be resolved at the synchrotron through transmission based imaging using an unfocused beam. When combined, imaging can provide the structural basis for the larger scale structure that cannot be resolved using USAXS alone and extend the accessible size range to millimetres and above.Although this implementation may be difficult for pinhole instruments, Bonse–Hart instruments have already been utilized to perform imaging within the crystal rocking curve [diffraction-enhanced imaging (Chapman *et al.*, 1997 ▸)] or outside the rocking curve [USAXS imaging (Levine & Long, 2004 ▸)]. Although the reconstruction of DEI is mature due to its simpler refraction based physics (Dilmanian *et al.*, 2000 ▸), reconstruction from USAXS imaging is not straightforward and still requires theoretical effort (Zhang *et al.*, 2008 ▸). Nevertheless, combining USAXS and imaging (including tomography) represents a logical next step for USAXS instrumental development, and the possibility should be explored.

Synchrotron X-rays also have the energy tunability that enables elemental sensitivity. Although anomalous (U)SAXS is not a possibility for fixed-energy beamlines, its potential should be considered elsewhere. For example, if multiple types of precipitates of similar sizes develop within an alloy, their small-angle scattering profiles will overlap. Anomalous scattering allows these precipitates to have different contrasts, and by monitoring the systematic change of the scattering intensity near an absorption edge, the size distribution of these precipitates can be separated. Note that combined USAXS and spectroscopy measurements, while rarely done, have value for understanding changes in materials chemistry, with apparent applications in catalysts and sustainability studies.

The discussion of multimodal measurements extends beyond X-ray based techniques. The previously mentioned combination of neutron and X-ray scattering in the ultra-small-angle scattering regime has significant practical implications for research on hard materials (Ohnuma *et al.*, 2009 ▸), and we encourage both researchers and funding agencies to consider this. Additionally, a variety of analytical techniques, such as mass spectrometry, calorimetry and digital image correlation can be integrated into synchrotron measurements to support *in situ* experiments using USAXS. We strongly advocate for continued investment and development of these capabilities at synchrotrons to maximize their potential.

### 5.2. Coherence

One hallmark of fourth-generation synchrotron sources is their significantly improved coherent flux (Einfeld, 2014 ▸), often by an order of magnitude or more at energies greater than 20 keV. This enhancement extends the applicability of coherence based techniques to energies up to 50 keV. In the USAXS regime, coherent scattering enables the investigation of structures and fluctuations in materials across length scales from tens of nanometres to several micrometres. This opens possibilities for investigating hierarchical and complex structures and dynamics that are often inaccessible by other means.

Coherent scattering techniques, such as XPCS, significantly benefit from improved coherent flux. In the context of XPCS, this enhancement allows for faster data acquisition and, consequently, the observation of faster dynamics. Note that XPCS in the ultra-small angle regime has been developed separately and successfully applied to both Bonse–Hart USAXS instruments (Zhang *et al.*, 2011 ▸) and pinhole USAXS instruments (Chèvremont *et al.*, 2024 ▸). When all factors are considered equal, the pinhole USAXS instrument based XPCS has an inherent advantage with its simultaneous multi-speckle data acquisition, which provides much better statistical sampling compared with its Bonse–Hart counterpart. Such multi-speckle measurements are particularly beneficial for materials with heterogeneous dynamics, allowing localized physical events to be investigated with greater precision (Andrews *et al.*, 2018*a* ▸,Andrews *et al.*, 2018*b* ▸). The advantage of the Bonse–Hart version of the XPCS resides in its possibility to access higher energies (>20 keV) without the need for building long instruments. For hard materials, XPCS analysis can yield valuable insights regarding domain fluctuation, grain growth, pore formation and relaxation dynamics upon stimulus. This capability has had limited exploration, with ample research opportunities across a broad spectrum of materials science.

### 5.3. Time-resolved study

Bonse–Hart and pinhole USAXS instruments face different challenges in achieving their respective resolution limits. Bonse–Hart instruments utilize point-scanning, making the motor speed a limiting factor. Using highly specialized, high-speed and high-precision motors, along with adopting fly scan, one can maximize data acquisition rates. However, conducting a precise USAXS scan under 15 s, or a combined USAXS–SAXS–WAXS measurements using a Bonse-–Hart instrument in under 30 s, remains challenging.

The time resolution of pinhole instruments is constrained by the material’s scattering power, beam flux, detector setup as well as the detector’s efficiency. Millisecond time resolution is currently achievable in certain cases. Nonetheless, note that, generally, the transformation kinetics of a material are size-dependent: the larger the size, the slower the required time resolution. For most of the material systems discussed here, a time resolution of a minute is sufficient to gather the necessary data to interpret physical processes in the size regime relevant to USAXS.

### 5.4. Machine learning and autonomous experiments

Though some practitioners of USAXS may recall using floppy drives, modern synchrotron X-ray facilities have evolved into significant sources of scientific data. It is not unusual for a two-day beam time at a Bonse–Hart instrument to produce tens of gigabytes of scattering data. For pinhole based USAXS instruments, which generally have larger detectors and faster acquisition rates, the data volume can exceed one or two orders of magnitude.

The effective use of synchrotron data presents a challenge for synchrotrons worldwide, across various techniques. Machine learning emerges as one of the most effective tools for efficiently analyzing and interpreting X-ray data. Its capability to detect patterns and trends has been utilized in numerous techniques such as X-ray imaging (Dixit *et al.*, 2020 ▸); diffraction (Zhao *et al.*, 2023 ▸); and, in certain cases, SAXS (Tomaszewski *et al.*, 2021 ▸). Quantitative SAXS data analysis poses a fundamental challenge for machine learning due to its model-dependent nature: a single scattering curve can theoretically have an infinite number of solutions. Nonetheless, machine learning has shown its strength in data classification, as illustrated by recent examples that integrate Gaussian processes and stochastic gradient descent methods (Archibald *et al.*, 2020 ▸). We also see the value in implementing automated general-purpose analysis tools, like the Unified Model, with trained machine learning models to provide an initial insight into the data. However, we recommend using results with these methods directly and indiscriminately with caution because all SAS analysis methods introduce an assumption bias. Meaningful SAXS analysis requires construction of a model based on known prior information.

In USAXS–SAXS–WAXS analysis, leveraging complementary datasets can enhance analysis. For instance, the nucleation and growth kinetics of precipitates are indicated by the evolution of their SAXS signature, including Guinier and Porod components. Simultaneously, the formation of a new crystalline phase is marked by the appearance and growth of diffraction peaks. These SAXS and XRD signatures must align kinetically, offering a chance for methods like principal component analysis to distinguish scattering profiles from different phases. With development, this type of across-the-range analysis provides an opportunity to gain insights into the microstructure formation process during *in situ* experiments.

The rapid classification and analysis by machine learning also enable autonomous experiments (Fukuto *et al.*, 2023 ▸), which have proven valuable in materials discovery, especially in combinatorial materials research. When applied to USAXS, this approach can help to identify optimal microstructures rather than phases. Moreover, the microstructure formation in many engineered materials is driven by kinetics rather than thermodynamics, making it pathway-dependent. Autonomous experiments offer a cost-effective way for process optimization.

The successful implementation and application of machine learning and autonomous experiments in the USAXS regime are currently very limited. However, we believe that these methods will increasingly become an essential component of USAXS operations and drive the advancement of hierarchical materials.

## 6. Conclusions and outlook

In this paper, we have critically reviewed the significant advancements and applications of USAXS in the context of hard materials science. Hard materials, with their superior strength and diverse functionality, have enormous practical significance and economic value. Their properties ubiquitously originate from their hierarchical structures across continuous length scales from sub-ångstrom to micrometres and above. Structural characterization over such extensive length scales represents a significant challenge and need. The development and application of USAXS, alongside complementary techniques such as SAXS and WAXS has opened new avenues to meet this challenge, both *in situ* and ex situ, for investigating the microstructural characteristics, structural hierarchy and kinetic transformations of hard materials under external stimuli. The knowledge gained from these measurements has proven useful in enhancing our understanding of a wide range of materials, from alloys and ceramics to cement, geological materials, and advanced technological materials.

Through its long development history, USAXS primarily serves as a niche technique reserved for special applications. The instrumentation for USAXS in the high-energy X-ray regime is challenging, making the footprint and impact of USAXS in hard materials science limited. However, in recent decades, USAXS instruments have seen rapid development worldwide, aided by the demonstrable success from selected USAXS instruments and the significant advancement in accelerator and detector technologies, as small angle scattering is a photon-starving technique with its intensity decaying following a steep power-law slope to the magnitude of the reciprocal vector.

These new developments of USAXS instruments, especially those capable of high-energy X-ray scattering, empowers USAXS to generate a bigger impact for hard materials science. We have carefully reviewed the instrumentation needs and challenges of two primary types of USAXS instruments: pinhole instruments and Bonse–Hart; evaluated their respective strengths and weaknesses; and identified opportunities for their continued development. The instrumentation selection criteria we establish in this review are subjective and limited by our knowledge and perspective. However, we hope these ideas will serve as stepping stones for continued instrumentation development and success.

We note that the full potential of USAXS is yet to be realized, primarily due to its underutilization in the hard materials sector. This can be attributed to a lack of familiarity with the technique’s capabilities within the scientific community. Contrary to common belief, ‘If you build it, they will come,’ we argue that continuous outreach and education are necessary to bridge this gap and to ensure more scientists and engineers can leverage USAXS for their research that requires detailed characterization of materials from atomic structure, mesostructure, to microstructure. This is also one of the main purposes of this paper.

We believe that the future of USAXS in hard materials science is bright, with ongoing technical developments at the instrument level and the advancements of fourth-generation synchrotron sources that offer much-enhanced brightness and coherence. These developments, especially with the improved characteristics of hard X-rays, will provide even deeper insights into the dynamic behaviors and structural transformations of hard materials. We are also excited about the potential of the integration of machine learning and autonomous experimental approaches with USAXS measurements. The unique measurement capabilities and more efficient analysis will unleash the potential of USAXS to lead to and support more discoveries in hard materials science.

## Footnotes

1 Certain commercial products or company names are identified here to describe our study adequately. Such identification is not intended to imply recommendation or endorsement by the National Institute of Standards and Technology, nor is it intended to imply that the products or names identified are necessarily the best available for these purposes.
